# Cochlear Implantation in Obliterated Cochlea: A Retrospective Analysis and Comparison between the IES Stiff Custom-Made Device and the Split-Array and Regular Electrodes

**DOI:** 10.3390/jcm11206090

**Published:** 2022-10-16

**Authors:** Julia Anna Christine Hoffmann, Athanasia Warnecke, Max Eike Timm, Eugen Kludt, Nils Kristian Prenzler, Lutz Gärtner, Thomas Lenarz, Rolf Benedikt Salcher

**Affiliations:** 1 Department of Otorhinolaryngology, Head and Neck Surgery, Hannover Medial School, 30625 Hannover, Germany; 2 Cluster of Excellence “Hearing4all”, Hannover Medical School, 30625 Hannover, Germany

**Keywords:** inner ear, cochlear implant, obliteration of the inner ear, ossification, fibrous tissue growth, electrode impedance, insertion probe

## Abstract

Anatomical malformations, obliterations of the cochlea, or re-implantations pose particular challenges in cochlear implantation. Treatment methods rely on radiological and intraoperative findings and include incomplete insertion, the implantation of a double array, and radical cochleostomy. In addition, a stiff electrode array, e.g., the IE stiff (IES) custom-made device (CMD, MED-EL), was prescribed individually for those special cases and pre-inserted prior to facilitate cochlear implantation in challenging cases. Data on outcomes after implantation in obliterated cochleae are usually based on individual case reports since standardised procedures are lacking. A retrospective analysis was conducted to analyse our cases on obliterated cochleae treated with MED-EL devices in order to allow the different cases to be compared. Impedances and speech perception data of patients treated with the IES CMD and the double array were retrospectively compared to patients treated with a STANDARD or FLEX electrode array (the REGULAR group). Patients with a Split-Array CMD had a poor speech perception when compared to patients treated with the IES CMD device. Thus, the IES CMD can successfully be used in patients with obliterated cochleae who would otherwise be non-users, candidates for a Split-Array CMD, or candidates for partial insertion with insufficient cochlear coverage.

## 1. Introduction

Hearing loss and deafness are associated with severe consequences for the affected patients, such as insufficient speech development, anxiety, depression, as well as lower educational and career opportunities due to social isolation [1,2] and an increased risk for the development of dementia [3]. Thus, the early diagnosis and treatment of hearing loss has a high socio-economical value.

Patients with severe sensorineural hearing loss are treated with a cochlear implant (CI) [4]. The surgical technique and the insertion technique are largely standardised, and regular CI electrodes can be inserted in the majority of cases. Special cases, however, such as the implantation of patients with anatomical malformations, obliterations of the cochlea, or re-implantations pose a challenge in cochlear implantation and may require special devices. Obliteration of the cochlea, for example, occurs after meningitis, trauma, or infection which result in hearing loss and subsequent intracochlear tissue growth, such as connective tissue or bone formation. When the cochlea is obliterated or ossified to a particularly significant degree, the conventional insertion of the mechanically flexible electrode array may be impossible [5], and alternative surgical techniques, such as incomplete insertion, the implantation of double arrays [6], implantation into the scala vestibuli [7,8], or a radical cochleostomy, must be considered. All these methods are associated with some disadvantages, such as the poor performance of the implant. However, despite a significantly higher risk for injuring the facial nerve, the internal carotid artery, or the modiolus [9], such alternative procedures are recommended in cases of partial and complete ossification.

To enable insertion even in cases with abnormal cochlear anatomies, special electrode arrays have been developed. MED-EL (Innsbruck, Austria), for example, created a compressed array as well as a Split-Array CMD for special requirements. The compressed array features 12 pairs of electrodes with an active stimulation range (ASR) of 12.1 mm (standard array ASR 31 mm), allowing the array to be placed in close proximity to the neurons, especially in malformed or partially ossified cochleae [10]. The split electrode array (MED-EL) with a double-branch electrode array was designed for the severe ossification of the cochlea. It contains five and seven electrode pairs on separated arrays on an ASR of 4.4 and 6.6 mm, respectively, which can be inserted through two cochleostomies (one in the basal and the second in the medial part of the cochlea) to increase the number of completely inserted electrode contacts in the ossified cochlea. Nevertheless, it can be shown that the speech performance of patients treated with this type of electrode is in the lower range of the spectrum that can be achieved by patients with a regular cochlear anatomy implanted with MED-EL’s STANDARD and FLEX electrodes [10,11].

Since August 2015, some of those challenging cases were also treated with a custom-made device (CMD, MED-EL) in our clinic. The CMD comprises a stiff insertion electrode (insertion electrode stiff, IES) and was prescribed in individual cases when the patients received a flexible lateral wall electrode array and there is an obstruction of the scala tympani (e.g., due to fibrosis or ossification). In such cases, the device is inserted prior to electrode insertion to dilate the cochlear lumen. This retrospective analysis evaluates speech performance data and impedance values as well as postoperative symptoms of patients treated with the IES CMD and compares them to the current available treatment options, a Split-Array CMD or a normal insertion.

## 2. Materials and Methods

### 2.1. Patients

A retrospective analysis of all patients treated between August 2015 and March 2019 with a MED-EL device revealed 33 patient ears which were treated with lateral wall CI electrodes using the IES CMD prior to electrode insertion (the IES group). Demographic and clinical data, impedance values, hearing results, and speech performance results of the patients were collected retrospectively. We also retrospectively identified patients treated with the STANDARD or FLEX electrode arrays to be included in our analysis as part of a comparative control group (the REGULAR group). The REGULAR group consisted of patients selected to match the patient’s age (±5 years), the patient’s gender, the electrode carrier, and the type of implantation (first implantation or re-implantation) of the IES group. As an additional control, we identified and included patients treated with a Split-Array CMD (the SPLIT group). In these patients, implantation with regular electrode arrays was impossible. Due to the special electrode carrier, there were no suitable match patients for the SPLIT group to form a comparative collective.

### 2.2. Study Design

Based on the retrospective design of the study, ethical board approval was not required. The data for the present analysis were extracted from our cochlear implantation database. This database was established to routinely collect all clinical, audiological, radiological, and surgical data of the patients. We retrospectively identified patients, in whom either a IES CMD or a Aplit-Array CMD was used for cochlear implantation. In addition to the data obtained from the database, we also collected data retrospectively from the patients’ surgical reports to determine the indication criteria used to determine the electrode type in individual cases. After identifying these patients, measurement protocols for impedances and hearing tests were evaluated, as well as clinical data collected from patient records to ensure a comprehensive evaluation. The REGULAR group served as a control group and included routinely treated patients without any cochlear abnormalities, as radiologically and intraoperatively determined.

### 2.3. The Fibrotic Obliteration Probe

**Figure 1:**
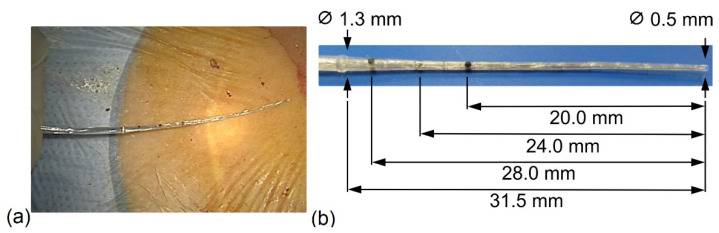
Dimensions of the IE stiff CMD. (**a**) Intraoperative; (**b**) markings on the IES CMD array. The IES CMD has a maximal insertion length of 31.5 mm.

The IE stiff CMD (IES, MED-EL, Innsbruck, Austria) (Figure 1) is a custom-made device which can be used individually when fibrotic tissue is present in the inner ear. It is used for the dilation of the cochlear lumen prior to electrode insertion. Thus, the IES consisted of an electrode dummy, which was used prior to electrode insertion as a surgical tool with no electrical function. The outer geometry was the same as the distal 50 mm of MED-EL’s STANDARD electrode, with a diameter of 1.3 mm at the proximal end and 0.5 mm at the distal end of the array (Figure 1a,b). It was made of medical-grade silicone with multiple stiff metal wires incorporated into its matrix. The markings on the IES CMD array at 20, 24, and 28 mm indicate the possible insertion depths (Figure 1b).

### 2.4. The Surgical Procedure

Electrode insertion via the round window is the preferred approach in cochlear implantation. After opening the round window for insertion, the IES CMD was carefully inserted into the inner ear for the dilatation of obstructed scala tympani or for depth measurement prior to the actual insertion of the electrode array in the IES group. In any case, the electrode array was inserted slowly into the scala tympani up to the previously defined insertion depth. The insertion site was sealed with muscle fascia from the temporal muscle after electrode placement. The array was usually fixed in a 1 mm bony notch drilled in the chorda facial angle. At the end of the surgical intervention, cone beam computed tomography (CB-CT) was performed to assess the intracochlear position of the electrode array.

In cases of ossification of the round window region or of ossification commencing at the basal turn, cochleostomy was performed in order to insert the electrode array. Therefore, the opening of the membrane was extended in an antero-inferior direction with a bur.

In order to supply the severe ossification patients with a split electrode array, superior cochleostomy at the level of the second turn was performed for the full insertion of the apical array (the SPLIT group).

### 2.5. Impedance Measurement

The impedance values were measured with the MED-EL telemetry system (MAX interface box, clinical software Maestro), enabling an impedance field telemetry (IFT) on all 12 electrode contacts. The used stimuli were biphasic pulses (24.2 µs) with a nominal amplitude of 300 current units (cu), where 1 cu approximates 1 µA [12]. The measurements were carried out at defined points after surgery as follows. The first measurement was taken during surgery (intraoperatively, after electrode insertion). The second measurement was taken at the end of the first fitting (FF), usually 4–10 weeks after surgery, or sometimes longer in rare exceptional cases due to previous complications. The following measurements were performed 3, 6, and 12 months (±4 weeks) after the FF. Until the FF, the implant was not activated since there was no audio processor worn by the patient, meaning that there was no electrical stimulation. After the first fitting, the implant was activated and the cochlea was electrically stimulated on a daily basis.

### 2.6. Statistical Analysis

For the statistical analysis, the impedance values of all 12 electrode contacts of a patient were averaged at one time point to obtain a mean value. The one-way ANOVA and post-hoc Tukey HSD multiple-comparison tests were used to compare the means and calculate the *p* values using IBM SPSS Statistics 26.0. Differences were considered statistically significant for *p* values < 0.05. All data were visualized using GraphPad Prism Version 8.3.0 and Microsoft Excel.

### 2.7. Hearing Tests

In order to evaluate speech understanding after cochlear implantation, the Freiburg monosyllabic speech test (FMB) and the Hochmair–Desoyer–Schulz–Moser (HSM) sentence tests [13], both in quiet and in noise at 0° azimuth (S0N0) and at a 10 dB signal-to-noise ratio (SNR), were performed in our clinical routine and evaluated retrospectively. All speech tests were conducted in the free field or were directly coupled with sentences and monosyllables presented at 65 dB SPL [14]. The measurements were carried out at the same time points as the impedance measurements.

### 2.8. Clinical Data

Clinical data were collected from the patients’ medical history and routine measurements retrospectively. For example, prior to and after implantation, patients were asked whether they suffered from vertigo, tinnitus, and facial stimulation.

### 2.9. Ethical Statement

This research is based on data collected from a retrospective analysis. Upon admission, all patients or their legal representatives signed an informed consent with regards to the anonymized use of their data for research purposes.

## 3. Results

### 3.1. Patients

Patients who underwent implantation with a MED-EL cochlear implant and who used the IES CMD prior to the insertion of the electrode array were included in the IES-group. Patients with different types of electrodes were included: FLEX 16 CMD (*n* = 1), FLEX 20 (*n* = 3), FLEX 24 (*n* = 3), FLEX 28 (*n* = 21), and STANDARD (*n* = 5).

Of these patients, twenty-seven were treated unilaterally and three were implanted bilaterally. In the following, each implanted ear is considered individually, yielding a dataset of 33 ears with nearly equal sex distribution (17 male and 16 female). Of the implantations, 20 (60.6%) were performed on the right side and 13 (39.4%) on the left side. At the time of implantation, the mean age was 37.6 years (range: 10 months–80 years).

The SPLIT group consisted of eight patients (five male and three female; mean age at the time of implantation 38.5 years; range: 1–74 years) who were implanted with a Split-Array CMD. In this group, the IES CMD was not used or its application was unsuccessful in the previous intervention (*n* = 3).

Both groups, the IES group and the SPLIT group, consisted of diverse and partly multimorbid patients. Table 1 provides an overview of the relevant comorbidities of the subjects as listed in their medical records.

For better comparability with various CI patients, for whom no IES CMD application was necessary or indicated, the REGULAR group was included. The REGULAR group consisted of patients who were age- (±5 years) and gender-matched (17 male and 15 female) to the subjects of the IES group. For the implanted patients of this group, the distribution of electrode variants was identical to the IES group. Only the implantation with a Flex 16 electrode array was excluded from the REGULAR group as no suitable comparison case was identified. This resulted in a dataset of 32 subjects for the REGULAR group. Table 1 shows the concomitant diseases of the REGULAR group to provide a complete survey.

### 3.2. Aetiology of Deafness

Regarding the aetiology of deafness in the IES group, seven patients (21.2%) suffered from meningitis, eight (24.2%) suffered from anatomical malformations in the inner ear, five (15.2%) suffered from other congenital hearing impairment, two (6.1%) suffered from sudden hearing loss, one (3.0%) suffered from trauma of the temporal bone, one (3.0%) suffered from otosclerosis, and the aetiology was unknown or not documented in nine other patients (27.3%).

In the SPLIT group, four patients (50%) suffered from meningitis, two (25%) suffered from trauma of the temporal bone, one (12.5%) suffered from sepsis due to pneumonia, and the cause was unknown or not documented for one other patient (12.5%).

### 3.3. Indication of the IES CMD

The IES CMD was used in our clinic for different indications. The possible applications included the penetration into a cochlea obstructed with fibrotic or ossified tissue, which could not be achieved by common electrodes due to their lack of stiffness and their inability to dilate the lumen. It was also used as an aid in re-implantations. Furthermore, the IES CMD was utilised as an instrument to estimate the depth of possible electrode insertion in the malformed cochleae.

Thus, the IES was deployed in the evaluated IES group to measure the depth of the cochlea prior to implantation (*n* = 7; 21.2%), due to intracochlear tissue which hampered the insertion of the electrode array (*n* = 10; 30.3%), as well as to ossify the basal turn or the round window region (*n* = 9; 27.3%). The IES was also used four times (12.1%) as a tool during device re-implantation to clear out fibrotic tissue. In three cases (9.1%), the reason for using the IES was to eliminate any resistance that occurred when trying to insert the stimulation electrode.

In the evaluation above, a variable was disregarded, depending on whether the surgical intervention was a first implantation or a re-implantation of a CI. In the IES group, 22 subjects (66.7%) underwent first implantation with an average duration of hearing loss of 8.57 years (range: 1 month–31 years). Eleven cases (33.3%) underwent re-implantation surgery due to soft failure or malfunction of the implant. The previous implant was in situ for a mean of 9.82 years (range: 6 month–31 years).

In the REGULAR group, patients were matched to the IES group (*n* = 22 first implantations and *n* = 11 re-implantations). An exact adjustment to the duration of hearing loss or the duration of the previous implant in situ was not possible due to the limited number of patients available.

In the SPLIT group, six cases (75%) were provided with a Split-Array CMD during the initial implantation procedure and showed an average duration of deafness of 5.04 years (range: 6 month–24 years). Two cases (25%) received a Split-Array CMD during a re-implantation procedure after 25 years in the first case and after 1 year in the second case.

### 3.4. Impedances

**Figure 2:**
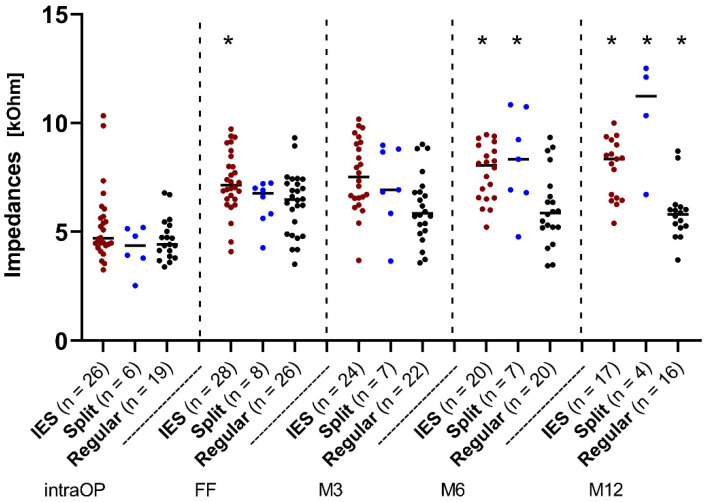
Change in impedances over time across the IES, SPLIT, and REGULAR groups. Median overall electrode contacts C1–C12. Since the number of patients varied over time, the existing patients were additionally marked as individual dots at each point in time. The red dots represent the IES patients, the blue dots represent the patients from the SPLIT group, and the black dots represent the patients from the REGULAR group. Asterisks mark the significant differences between groups.

The course of impedances over time is depicted in Figure 2. Here, the median values of the overall impedances of all electrode contacts of the IES and SPLIT groups were plotted and compared to those of the REGULAR group (Figure 2). The median values were almost equal between the three groups immediately after the insertion at the intraoperative measurement. Thereafter, an increase in the impedance values up to the first fitting (FF; 4–6 weeks after implantation) was observed in all groups, but at slightly different levels. In the IES group, impedances continued to rise with a slight linear increase up to 12 months after the FF, while in the REGULAR group, a slight decrease was observed and finally constant impedance values were measured at a much lower level than the IES group. The values of the SPLIT group stayed relatively stable on all contacts up to 3 months. Thereafter, a strong increase in impedance values up to 12 months was observed that went far beyond the measured values of the IES group. Statistically significant differences could be observed at the FF between the IES and REGULAR groups (*p* < 0.05) and at M6 between the IES/CONTROL group (*p* < 0.01) and the IES-/SPLIT group (*p* < 0.05). At M12, all three groups showed a significant difference to each other (IES/REGULAR, *p* < 0.001; IES/SPLIT, *p* < 0.05; SPLIT/REGULAR, *p* < 0.001).

#### 3.4.1. Impedances at Different Indications of IES Use in the IES Group

**Figure 3:**
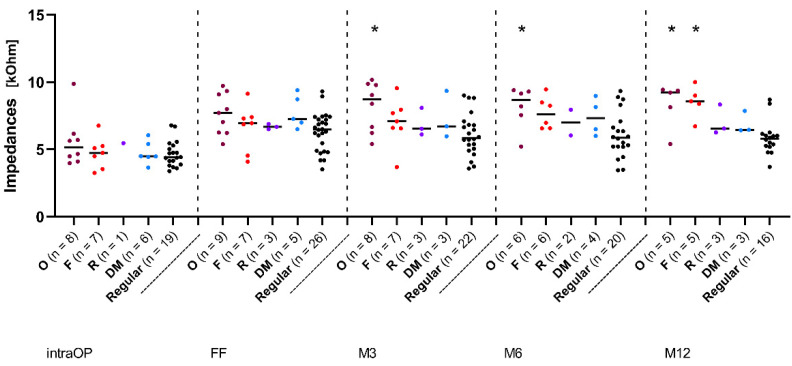
Change in impedances over time in subjects with different indications of IES use. Median overall electrode contacts C1–C12. The magenta dots represent patients with cochlear ossification (O), the red dots represent patients with fibrosis (F), the purple dots represent patients with occurring resistance (R), and the light blue dots represent patients for which the IES was used for cochlear depth measurement (DM). The black dots represent patients from the REGULAR group. Asterisks mark significant differences between groups.

In order to examine the diverse IES group in more detail, a retrospective subdivision into four subgroups, based on the indications for which the IES CMD was prescribed in the implanted ears and thus on the aetiology of the patients’ deafness, was performed. Figure 3 shows the impedance data over time of the subgroup, where the IES CMD was applied due to an existing ossification in the inner ear (magenta dots, the O group, Figure 3), as well as the subgroup with IES CMD application due to fibrosis in the inner ear (red dots, the F group, Figure 3). The third subgroup indicates that IES CMD use after a resistance occurred during the actual implantation of the stimulation electrode (purple dots, the R group, Figure 3). In the fourth subgroup, the IES was used to determine the depth of the cochlea (light blue dots, the DM group, Figure 3). To facilitate a constant comparison, the REGULAR group was also depicted here with impedance data over time (black dots, Figure 3).

Figure 3 shows the median impedance values over time over all electrode contacts. From the FF onwards, there was a slight drop in the impedance values and finally a constant lower level in the REGULAR group, while all other groups continued to show slight impedance increases. The O group showed the highest values over the whole time, followed by the F group. The R and DM groups, on the contrary, were at almost the same median level, but showed slightly higher values than the REGULAR group. From month 3 (M3) onwards, there was a statistically significant difference between the O group and the REGULAR group (*p* < 0.05). After 12 months, the F group also showed significantly higher impedance values than the REGULAR group (*p* < 0.01). All other groups showed no significant differences at any time.

#### 3.4.2. Impedances Re-Implantation vs. First Implantation

In order to evaluate whether the re-implantation procedure and the usage of the IES CMD has an influence on impedance development, we distinguished between the cases of first implantation and the cases of re-implantation in both the IES group and the REGULAR group, as follows:

1. Re-implantation in the REGULAR group (RR): consists of 10 patients who received a re-implantation without using the IES;
2. Re-implantation in the IES group (RI): consists of 11 patients who were re-implanted using the IES;
3. First implantation in the IES group (FI): consists of 22 patients who were implanted for the first time using the IES;
4. First implantation in the REGULAR group (FR): consists of 22 patients who were implanted for the first time without the IES.

**Figure 4:**
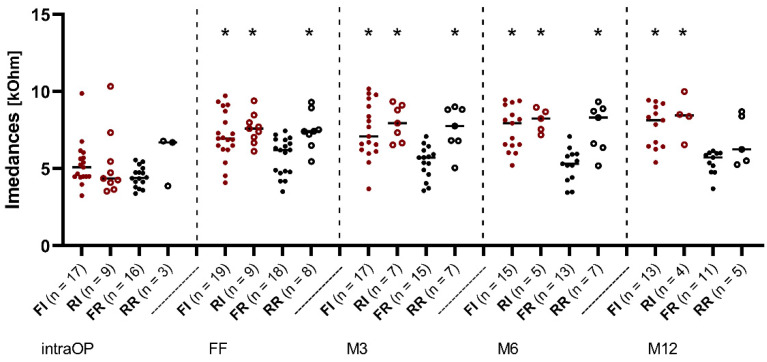
Change in impedances over time re-implantation vs. first implantation in the IES and REGULAR groups. Median over all electrode contacts C1–C12. The filled red dots represent the patients from the IES group who received first implantation (FI) and the hollow red dots represent the patients from the IES group who underwent re-implantation (RI). The filled black dots represent the patients with first implantation from the REGULAR group (FR) and the hollow black dots represent the patients who underwent re-implantation from the REGULAR group (RR). Asterisks mark significant differences between groups.

The course of median values over all electrode contacts over time is presented in Figure 4. Up to the FF, there was a similar increase in impedance values in all groups. In the further course, the median values of the RR, RI, and FI groups increased, while a decrease in the impedances could be observed in the FR group. Statistically significant differences between the FR and FI groups (*p* < 0.05), FR and RI groups (*p* < 0.01), and FR and RR groups (*p* < 0.05) could also be observed, which remained until month 6 (M6). After 12 months, there was no longer any significant difference between the FR and RR groups.

### 3.5. Speech Comprehension

Due to the retrospective analysis and the resulting limited availability of datasets, both patients whose speech tests were conducted in the free field as well as in direct coupling were included in the following speech data evaluation.

**Figure 5:**
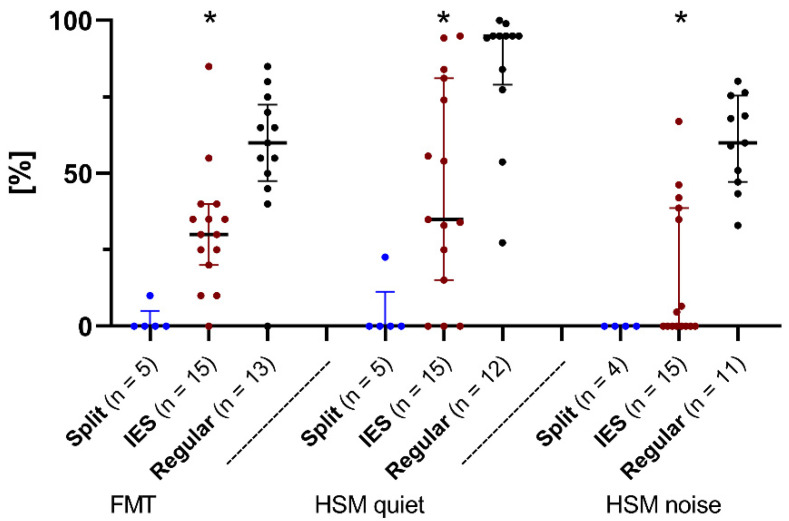
The Freiburg monosyllabic word test (FMT), and the HSM sentence test in quiet and in noise. Median scores (in % correct) after 6 months of device use. Comparing patients from the IES group (red dots), the REGULAR group (black dots), and the SPLIT group (blue dots). Asterisks mark significant differences between groups.

To avoid bilateral benefits, based on the results of the audiogram, an additional measurement in direct coupling was carried out if it was suspected that the non-test ear was influenced. Therefore, it can be assumed that the influence of the non-test ear was selected entirely from the speech results (Figure 5).

For the SPLIT group, only a few datasets were available since not every patient could complete each of the tests. As a consequence, the number of subjects varied between tests at measurement point after 6 months (M6). Furthermore, the results of a bilaterally implanted child in the SPLIT group were excluded from an evaluation of the speech data, as speech development was not possible due to child’s age. The exclusion allowed an unbiased trend of hearing performance in patients implanted with a split-array CMD.

After 6 months of device use, for FMT, the subjects belonging to the IES group scored a median of 30%. The median was 60% after 6 months in the REGULAR group and 0% in the SPLIT group. There were significant differences between the IES and REGULAR groups (IES/REGULAR *p* < 0.01). Due to the small size of the test group, no statistical evaluation of the SPLIT group was carried out.

For the HSM test in quiet, the subjects of the IES group scored a median of 34.9% after 6 months of device use. In comparison, the median values of the REGULAR group were 95% after 6 months and 0% after 6 months in the SPLIT group. An intergroup comparison between the IES and REGULAR groups showed a significant difference in the results of the HSM test in quiet (*p* < 0.01).

For the HSM test in noise, patients of the IES and SPLIT groups achieved median values of 0% after 6 months of device use, as opposed to the REGULAR group (63.49%; *p* < 0.001).

### 3.6. Clinical Data

The clinical data presented here are based on a retrospective analysis of the patients’ medical history before and after cochlear implantation. The numbers of patients suffering from tinnitus, vertigo, or facial stimulation at the respective times are shown in Table 2. Furthermore, seven patients in the IES group and two patients in the SPLIT group suffered from meningitis before surgery. None of the patients in the REGULAR group had a pre-existing meningitis in their medical history. After surgery, there was no evidence of postoperative meningitis in all three groups.

In order to alleviate facial stimulation in affected patients, triphasic pulses were utilized in four of the affected cases (Table 2), but were only temporarily sufficient for one of the cases. In the three remaining patients, individual electrode contacts had to be switched off and the stimulation level was lowered below the facial nerve stimulation (FNS) threshold in order to remedy the symptoms. The remaining two cases were not further documented because one patient was lost for follow-up (patient in the SPLIT group) or the other patient was currently no longer fitted with a CI (patient in the IES group).

## 4. Discussion

To our knowledge, this was the first comparative yet retrospective analysis to show that the IES CMD can facilitate the implantation of flexible lateral wall CI electrodes in patients with fibrotic, ossified, and malformed cochleae. Moreover, we showed that individual treatment with the IES CMD allowed a regular electrode array to be implanted without significantly impairing the performance of the patients.

Specifically, intraoperative impedance values of all three test groups were equally high and a presumed increase from insertion up to the FF was also evident in all three groups (Figure 2). At the timepoint M12, it can be stated that the median values of impedances of the IES and SPLIT groups were significantly higher than the median value of the REGULAR group. However, in relation to this, the median value of the SPLIT group was also significantly higher than the median value of the IES group.

In the REGULAR group, no significant increase in the median impedance values over time can be observed. Thus, based on the impedance values, an increased trauma due to the electrode insertion seemed unlikely here [15,16,17]. In contrast, there was an increase in impedances over time in the IES and SPLIT groups. A correlation between the level of fibrotic material and the impedance levels was found in preclinical cochlear implant models consisting of guinea pigs [18]. It could be possible that increased tissue formation due to insertion trauma may account for the increased impedance values observed in the IES and SPLIT patients. The increase in the IES group could be due to an increased amount of fibrous tissue growth around the electrode. However, it must be critically questioned whether this deviation, especially in comparison to the REGULAR group, is solely due to the use of the IES during surgery and the additional microtraumas that may have arisen. Patients in the IES group, unlike patients in the REGULAR group, already had increased cochlear damage (fibrosis, ossification, and malformation) before implantation and this damage may also explain the different impedance values. Another hypothesis therefore could be that the initial fibrotic tissue formation prior to implantation continues afterwards and leads to higher impedances. Furthermore, increased surgical trauma, such as cochlear drilling, as performed in some patients, could also trigger inflammation reaction with new tissue formation accounting for the increase in impedances [19]. In addition, it has not been evaluated whether other traumatic events, such as scalar shift, occurred after implantation, and whether they influenced the data. However, no scalar shift was described in the regular radiological evaluations of the postoperative CT images.

Further typical changes in electrode impedances occurred with the onset of electrical stimulation. It has been reported that the impedances of intracochlear electrodes are lower after stimulation compared to the levels before stimulation onset [20,21]. After implantation, a cell [22] and passivation [23] layer accumulated on the electrode surface of inactive electrode contacts. This layer was disrupted by the onset of electrical stimulation and resulted in a decrease in impedances, as clinically observed. Within 3 months after starting the electrical stimulation (after the FF), a decrease in impedances in all three groups, i.e., the IES group, the SPLIT group, and the REGULAR group, was observed in the present study. This decrease might corroborate studies showing that chronical electrical stimulation may lower electrode impedances [24,25,26].

As a result, it must be questioned whether patients in the SPLIT and IES groups have a shorter or more irregular wearing time of the implant processor to M12 and thus a non-regular electrical stimulation, thus contributing to an increase in impedances. However, this parameter was not recorded in our study.

Significantly increased impedance values 12 months after the FF were demonstrated in the groups with pre-damage of the cochlea (O and F groups) when compared to the REGULAR group (Figure 3). Pre-damage of the inner ear might have a significant influence on the further development of the electrode environment after implantation, leading to increased impedance values. No significant difference was found between patients who used the IES as a depth probe (the DM group) and patients in the REGULAR group, indicating that the IES is rather less traumatic when used in non-pre-damaged ears.

This assumption is contradicted by impedance values measured 12 months after implantation (M12), as depicted in Figure 4. While the impedances between all groups were still intraoperatively equal, the impedances of the IES group (FI and RI) were significantly increased from the FF onwards, as well as the values of the RR group. At M12, the values of the RR group dropped again. It is therefore assumed that without the use of the IES, the impedances will return to lower values over time. Thus, the IES could account for higher impedance values over time and should not be routinely used as a depth sensor, as the electrode environment appears to recover better without the use of the IES.

When interpreting the results, the usefulness of impedance values needs careful consideration. It is well accepted that electrode impedances may be a useful biomarker of inner ear pathology after cochlear implantation [27] and low impedances are desirable to minimize battery consumption. As such, impedances represent a non-invasive measuring method for obtaining information about the environment between electrodes and the respective neural interfaces [28]. It is believed that changes in the electrode impedances are related to the formation of a fibrous tissue matrix around the electrode array [18,22,29,30]. Foreign body immune responses may help to encapsulate the electrode array in fibrous tissue within the first few weeks of implantation [20,31]. Clinical studies on patients treated with cochlear implants show that, in the days and weeks following implantation, electrode impedances increase, forming a plateau after 4–6 weeks in situ [22,32].

Foggia et al. describes an inflammatory or fibrotic reaction as a response to the electrode array in the cochlea that occurs with every implantation [20]. Both the acute tissue response immediately after implantation (due to the insertion trauma) and the delayed response as a host-mediated foreign body response caused by nearly all biomaterials may help to explain the observed increase in impedance values [20]. Interestingly, a recent study has shown that impedances values do not correlate with speech understanding [33]. Considering the results of speech comprehension of the different groups 6 months after implantation, the patients of the REGULAR group achieved fairly good speech performance. The results obtained are consistent with those taken from other studies [34]. For the SPLIT group, poor results for speech perception were observed in our study, as corroborated by other studies [10]. Degeneration in spiral ganglion cells is particularly high in patients whose cause of deafness is bacterial meningitis [35]. Since many patients in the IES group had preoperative meningitis in their medical history, it can be assumed that a reduced number of spiral ganglion cells is one of the reasons for lower speech comprehension scores between the IES group and the REGULAR group. Therefore, as an important observation based on our results, it can be concluded that patients with implantation of a long lateral wall electrode using the IES CMD prior to insertion experience significant benefits in terms of speech understanding, as compared to those who were fitted with a Split-Array CMD (Figure 5). However, the case of a child implanted bilaterally with split arrays, which was excluded from our speech comprehension data, also shows that a long-term evaluation is indispensable in order to be able to make further statements. The child did not show any measurable scores at time M6 of the speech comprehension data evaluation as the tests were not age-appropriate; however, measurable speech understanding has developed in the meantime. To conclude, if conditions permit, normal implantation with the aid of the IES CMD should be favoured over the implantation of a Split-Array CMD. Speech recognition after cochlear implantation is further dependent on the degree of spiral ganglion cell preservation.

Since there are other stiff electrodes available for cochlear implantation, questions surrounding the use of delicate electrodes, such as the FLEX series from MED-EL, arise since the recipients presented with an obliteration of the cochlea with no residual hearing. The FLEX electrode array series includes atraumatic devices with variably sized lengths up to 28 mm. As such, this series allows the length of the electrode array to be correlated with the size of the patient’s cochlea, thus adapting to variations in cochlear geometry. An advantage of flexible electrode arrays is the avoidance of pronounced trauma to the cochlea during electrode insertion. Since patients in the IES group have previous damage up to the anatomically complete obstruction of the cochlea, there is no residual hearing worth protecting. Thus, the use of flexible electrodes does not seem beneficial from this point of view. However, several studies, such as those by Buchmann et al. [36] or Büchner et al. [34], have shown that speech understanding after implantation significantly depends on the insertion depth of the electrode and that the insertion of longer electrodes allows better speech perception. With this in mind, the electrodes used represent a promising means of achieving better speech understanding in affected patients, not due to their flexibility, but because of their potential increased cochlear coverage. Moreover, the various malformations in the IES group that we evaluated are cases in which the insertion length can often only be determined intraoperatively. The ability to adjust the insertion length accordingly is essential.

Meningitis may occur more frequently in patients implanted with a CI either due to local infections or as a result of the actual surgical intervention. Other risk factors such as congenital or acquired anatomical defects, previous meningitis, or immunodeficiencies are described [37]. In the IES group, no postoperative meningitis occurred at the time of evaluation, although many of the risk factors apply to the evaluated group (Table 1). Furthermore, the IES CMD does not appear to increase the risk for meningitis. Vertigo and vestibular dysfunction may occur postoperatively after cochlear implantation [38]. Patients of the IES group already showed symptoms of this category preoperatively, and the number of affected patients slightly decreased postoperatively (Table 2). Here, it must be considered that the pre- and postoperatively affected patients do not necessarily correlate with one another. However, it seems that the use of the IES CMD does not result in a strongly increased trauma risk, since clinical symptoms and adverse events are to be regarded as indicators. Postoperative facial stimulation is observed in patients of all three groups. In the REGULAR and IES groups, facial stimulation could be controlled and improved by the use of triphasic pulses, while no change occurred in the SPLIT group (Table 2).

There are several limitations associated with the present study. First of all, the small number of patients included in this study limits the conclusions that can be drawn from the data. Additionally, the inhomogeneous patients, especially in the IES group, make it difficult to compare the data to the other study groups. Despite these limitations, we were able to demonstrate significant differences between groups and we could derive some important observations from our data.

## 5. Conclusions

In summary, this study shows that the IES CMD can successfully treat patients who would otherwise be non-users or would only be able to receive a split-array CMD or an insufficient number of inserted electrode contacts. The IES CMD offers a method to insert long flexible lateral-wall electrodes into the cochlea with a concomitant low risk of clinical complications.

The above evaluation also shows the broad applicability of the IES CMD, as it is a tool that can be used in almost all age groups and for a wide range of diseases. The IES CMD forms an important safe surgical aid for special cases, which does not greatly prolong surgical intervention and makes successful implantation possible.

Nevertheless, the IES CMD should not be applied as a standard instrument for cochlear implantation because its use leads to higher postoperative impedances, possibly due to a more invasive and traumatic implantation when compared to the FLEX arrays without the IES CMD. If possible, imaging techniques, or, more specifically, manufactured insertion electrodes, should be used to determine cochlear length, eliminating any negative influence on hearing results using the IES. In the future, it may be interesting to evaluate whether steroid administration via an inner ear catheter [15] can lead to a further reduction in impedances after implantation with the IES CMD. Furthermore, the IES CMD also provides a means of overcoming restrictions in the cochlea, allowing more electrode contacts to be inserted when only partial insertion would be possible without its use. The question around whether speech understanding can be improved also needs to be clarified further in future research endeavours.

## Figures and Tables

**Table 1:** A summary of the occurrence of relevant concomitant diseases in subjects of the IES, SPLIT, and REGULAR groups.

	IES Group	SPLIT Group	REGULAR Group
relevant comorbidities; *n* (%)	*n* = 33	*n* = 8	*n* = 32
hypertension	10 (30.3)	4 (50.0)	8 (25.0)
meningitis	7 (21.2)	5 (62.5)	0 (0)
syndromes, genetic malformations	5 (15.2)	0 (0)	5 (15.6)
tumor in the head area	3 (9.1)	0 (0)	0 (0)
cardiac arrhythmia	2 (6.1)	2 (25.0)	1 (3.1)
diabetes mellitus (type I)	0 (0)	0 (0)	1 (3.1)
diabetes mellitus (type II)	2 (6.1)	2 (25.0)	4 (12.5)
coagulopathy	2 (6.1)	1 (12.5)	1 (3.1)
pulmonary diseases	2 (6.1)	3 (37.5)	4 (12.5)
epilepsy	1 (3.0)	1 (12.5)	0 (0)
apoplexy	1 (3.0)	1 (12.5)	1 (3.1)
hyperlipoproteinemia	1 (3.0)	0 (0)	1 (3.1)
osteoporosis	1 (3.0)	0 (0)	0 (0)
craniocerebral injury, fracture of temporal bone	1 (3.0)	2 (25.0)	1 (3.1)
facial nerve paresis	1 (3.0)	0 (0)	0 (0)
depression	1 (3.0)	2 (25.0)	1 (3.1)
sepsis	0 (0)	1 (12.5)	0 (0)

**Table 2:** Clinical data.

Clinical Data; *n* ^1^ (%)	SPLIT Group	IES Group	REGULAR Group
**vertigo** preoperative postoperative			
	1 (12.5)	8 (24.2)	7 (25.0)
	1 (12.5)	7 (24.1)	4 (14.3)
**tinnitus** preoperative postoperative			
	5 (62.5) 4 (50.0)	12 (36.4) 4 (13.8)	9 (32.1) 6 (21.4)
**facial stimulation** preoperative postoperative after triphasic pulses			
	0 (0.0)	2 (6.1)	0 (0.0)
	2 (25.0)	4 (13.8)	2 (7.1)
	2 (25.0)	1 (3.4)	1 (3.6)

^1^*n* = 8 patients were evaluated in the SPLIT group; *n* = 28 patients were evaluated in the REGULAR group; and *n* = 33 and *n* = 29 patients were preoperatively and postoperatively evaluated in the IES group, respectively.

## Data Availability

The data presented in this retrospective analysis are available on request from the corresponding author. The data are not publicly available as they contain personal and sensitive patient data. All data are stored on the servers of Hannover Medical School, Department of Otorhinolaryngology.
